# A modified kidney-sparing portal vein arterialization model of heterotopic auxiliary liver transplantation increases liver IL-6, TNF-α, and HGF levels and enhances liver regeneration: an animal model

**DOI:** 10.1186/s12893-022-01726-5

**Published:** 2022-07-21

**Authors:** Jun Li, Jianjun Ren, JunJing Zhang, Xingkai Meng

**Affiliations:** 1 Department of Hepatobiliary, Pancreatic and Splenic Surgery, The Affiliated Hospital of Inner Mongolia Medical University, Huhhot, 010050 People’s Republic of China; 2 Department of General Surgery, Hohhot First Hospital, Huhhot, 010030 People’s Republic of China

**Keywords:** Acute liver failure, Portal vein arterialization, Liver regeneration, Heterotopic auxiliary liver transplantation

## Abstract

**Background and Aim:**

The success of partial donor liver transplantation is affected by the implantation site of the donor liver and the vascular reconstruction approach. We investigated the effects of different donor liver implantation sites and vascular reconstruction approaches on liver regeneration using a rat kidney-sparing heterotopic auxiliary liver transplantation model, with portal vein arterialization (PVA).

**Methods:**

Sixty male Sprague–Dawley rats underwent end-to-end anastomosis of the donor liver portal vein and the right renal artery stent (control group), or end-to-side anastomosis of the donor liver portal vein and the left common iliac artery (experimental group).

**Results:**

The experimental group had significantly lower plasma levels of alanine aminotransferase (ALT), aspartate aminotransferase (AST), total bilirubin, and cholinesterase than the control group (all, *P* < 0.05). The levels of tumor necrosis factor-α (TNF-α), interleukin 6 (IL-6), and hepatocyte growth factor (HGF) in the liver were significantly higher in the experimental group than that in the control group (all, *P* < 0.05). Hematoxylin and eosin (HE) staining of the liver tissue specimens indicated that the experimental group had greater hepatocyte regeneration compared to the control group.

**Conclusions:**

The modified kidney-sparing PVA model of heterotopic auxiliary liver transplantation is more conducive to liver regeneration with quicker return of liver function.

## Introduction

Liver transplantation has significantly improved the prognosis of patients with acute liver failure, and is recommended for patients with acute hepatic failure who are unlikely to have a spontaneous recovery [1]. However, donor liver shortage has severely limited liver transplantation for these patients [2, 3]. The liver has enormous regeneration potential, and acute hepatic failure patients who survive without liver transplant can expect a complete morphological and functional recovery of the liver [4, 5].

Total orthotopic liver transplantation (as described above) is a lifesaving option for patients with acute or fulminant liver failure [6, 7]. However, the procedure requires lifelong immunosuppression in order to prevent rejection of the graft. To overcome this limitation, auxiliary partial orthotopic liver transplantation was developed [5]. In this procedure, only a portion of the native liver is removed and replaced with a portion of healthy donor liver. The transplanted liver portion provides temporary liver function while the native liver that was left in-situ recovers [6, 7]. Once the native liver recovers, immunosuppression can be withdrawn [6, 7]. However, outcomes vary based on the donor liver implantation site, vascular reconstruction approach, and other variables [3, 6, 7]. In the situation of a poor portal vein (PV) that cannot be reconstructed and/or PV thrombosis, Neuhaus et al. [8] proposed PV arterialization (PVA) to reduce the risk of donor liver PV thrombosis.

Heterotopic auxiliary liver transplantation with PVA avoids extensive dissection of the native liver and graft, and leaves more liver mass for the bridging graft and native liver regeneration [9, 10]. Studies have explored various methods of liver transplantation with respect to implantation site and PV reconstruction, aimed at reducing the volume of donor liver required for successful transplantation and liver regeneration [2, 7, 11].

The purpose of this study was to examine the effect of different donor liver implantation sites and vascular reconstructions with PVA with respect to liver regeneration in heterotopic auxiliary liver transplantation. We used a rat model, and in brief: (1) The control group underwent end-to-end anastomosis of the donor liver PV and the recipient right renal artery stent, donor liver supra-hepatic caval vein suturing, and donor and recipient infra-hepatic caval vein end-to-side anastomosis; (2) The experimental group underwent end-to-side anastomosis of the donor liver PV and the recipient left common iliac artery, reconstruction of the hepatic artery, end-to-side anastomosis of the donor supra-hepatic and infra-hepatic venal vein and the recipient inferior vena cava (IVC) (the recipient right kidney was not removed in the experimental group).

## Materials and methods

### Animals

Sixty specific pathogen free (SPF) male Sprague–Dawley (SD) rats, weighing 210–260 g, were obtained from the Laboratory Animal Center, the Third Military Medical University, Chongqing, China. The rats were maintained in a temperature- and humidity-controlled environment with a 12-h light/12-h dark cycle. Rats were allowed free access to standard food and water, and underwent an overnight fast before the experiment. The rats were randomly assigned to the control group and the experimental group.

The control group underwent end-to-end anastomosis of the PV of the donor liver to the recipient right renal artery stent. Donor liver supra-hepatic caval vein suturing, and donor and recipient infra-hepatic caval vein end-to-side anastomosis, and choledochojejunostomy were also performed. In the experimental group an end-to-side anastomosis of the donor liver PV to the recipient left common iliac artery was performed. Reconstruction of the hepatic artery, end-to-side anastomosis of the donor supra-hepatic and infra-hepatic venal vein and the recipient IVC, and choledochojejunostomy were also performed.

The study protocol was approved by the ethics committee of the Inner Mongolia Medical University (Permit No.YKD2015102), and the study was carried out in accordance with the institutional and state regulations on the use and care of animals used for scientific studies. All methods are reported in accordance with ARRIVE guidelines (https://arriveguidelines.org) for the reporting of animal experiments.

### Establishment of the PVA model in the experimental group

#### Donor liver extraction

**Fig. 1:**
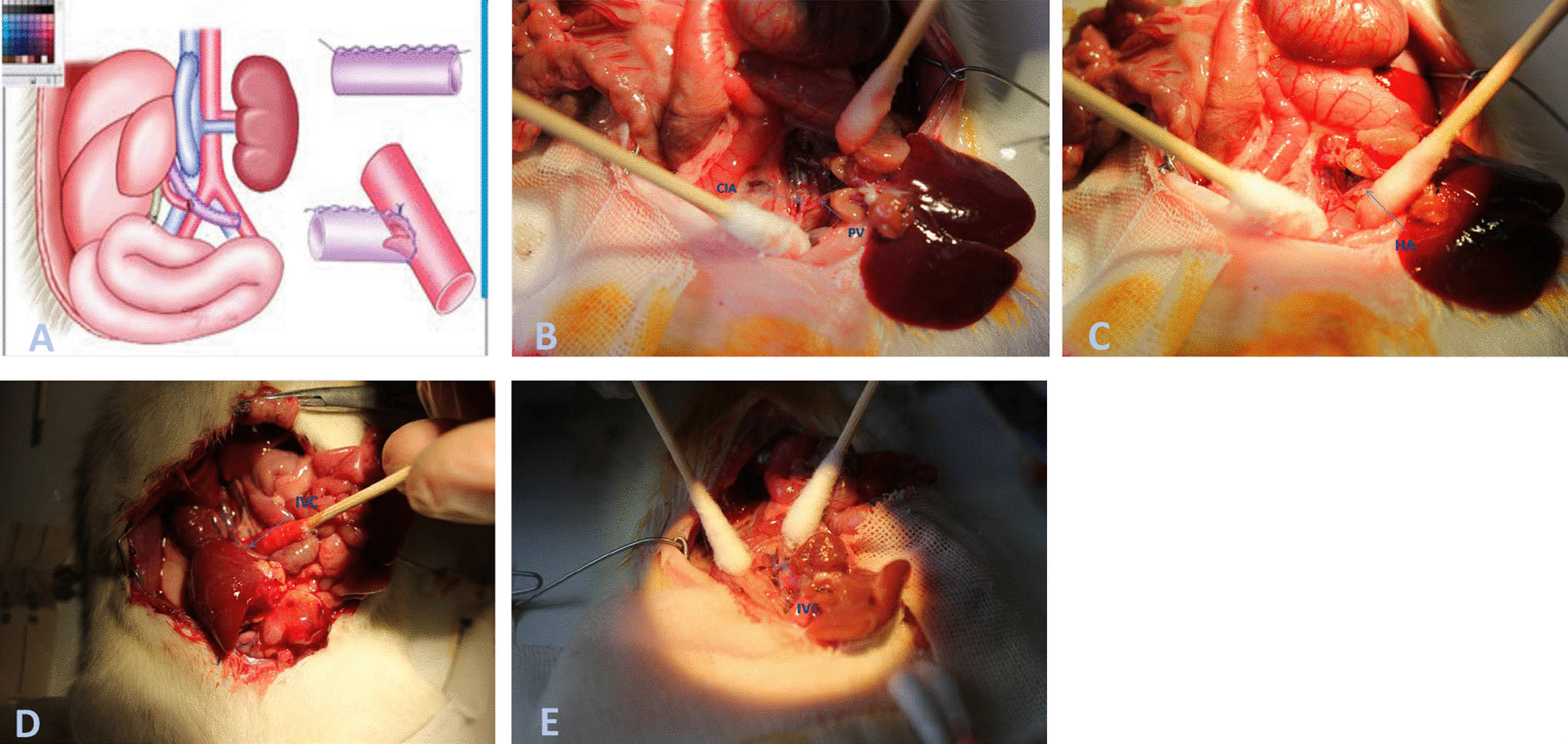
**A** The schema of heterotopic auxiliary rat liver transplantation with portal vein arterialization. **B** End-to-side anastomosis of the donor hepatic portal vein and the recipient left common iliac artery is made with a running 10–0 prolene suture. The free flap is embedded in the anastomosis to prevent an excessive blood flow speed or volume to reduce the chances of a high perfusion injury in the donor liver. **C**, **D** End-to-side anastomosis is made between the donor liver supra-hepatic and infra-hepatic caval vein and the recipient inferior vena cava. **E** End-to-end anastomosis is made between the right renal artery and the donor liver portal vein using the stent method

The schema of heterotopic auxiliary rat liver transplantation with PV arterialization is summarized in Fig. 1A. Operative procedures were performed using a surgical microscope with × 4–6 magnification. Rats were anesthetized with an isoflurane vaporizer (5% isoflurane) to induce anesthesia, which was then maintained with 1.5% isoflurane. The rats were placed in the supine position, shaved and sterilized, and 50 U of heparin was injected via the dorsal vein of the penis. A cruciform incision was made in the upper abdomen, and the infra-hepatic caval vein was dissected until the left renal vein was reached, without disturbing the liver. The right renal vein was ligated close to the inferior vena cava (IVC) with a 10–0 silk suture.

The PV was dissociated until the third tributary was reached. The first PV tributary was dissociated, and the second PV tributary was ligated. A wedge incision 2 mm inferior to the papillary process bile duct was made, a stent was introduced into the common bile duct, and a 10–0 double silk suture was used for ligation. The left gastric artery, the splenic artery, and the gastroduodenal artery were dissociated from the truncus coeliacus, and then ligated and transected; only the common hepatic artery was preserved. The abdominal aorta superior to the truncus coeliacus was dissociated, and a No. 1 stay suture was placed. Heparin (500 U) in saline was then injected into the infra-hepatic caval vein. A knot was made above the truncus coeliacus with the stay suture. The abdominal aorta above the knot was clamped, and the abdominal aorta below the knot was rapidly punctured, chilled to 4 °C, and a heparin saline solution was continuously infused via a micropump at 150 mL/h. The IVC was incised for outflow of the perfusate. The liver was continuously bathe with 4 °C normal saline, and the remaining liver was rapidly dissociated.

The caudate lobe dorsal to the greater curvature of the stomach was dissociated, and the venous plexus between the left liver and the esophagus and the left triangular ligaments were transected by cauterization. The phrenic vein was ligated close to the liver, and the diaphragm was incised in a circular fashion close to the vena cava foramen. The vena cava was dissociated 2–3 cm into the thoracic cavity and transected. Adhesions between the right lower lobe and the posterior peritoneum were dissociated and transected up to the triangular ligaments. The venous plexus of the right adrenal gland was dissociated, ligated, and transected close to the liver. The infra-hepatic caval vein was then transected at the level of the left renal vein, the first and second PV tributaries were transected, and finally the PV was transected at the level of the third PV tributary.

An oblique incision was made at the origin of the common hepatic artery, a hepatic artery stent was inserted, the vessel was ligated and fixated, and the common hepatic artery was transected proximal to the ligature and perfusion was discontinued. The donor liver was then removed and placed in a 10-cm dish containing chilled 4 °C heparin saline solution for trimming. Excess fatty tissues in the walls of the supra-hepatic and infra-hepatic caval vein and PV were removed, and the end to be used for anastomosis was trimmed. The common hepatic artery stent was anastomosed to the first PV tributary, ligated, and fixated with a 10–0 double silk suture in order to reconstruct the hepatic artery. The contralateral wall of the PV was sutured using 10–0 prolene in a running spiral fashion in the proximodistal direction, covering one third of the diameter of the PV. Approximately 70% of the lateral and medial segments of the left lobe were ligated using 9–0 silk suture and excised, and the remaining 30% was used as the donor liver and stored at 4 °C until use.

#### Recipient operation

A median laparotomy was performed, the xiphisternum was pulled cephalically using a large Bulldog clamp, and the intestines were covered with warm wet gauze. A 9–0 silk suture was used for ligation, and 30% of the left lateral lobe of the recipient liver was excised. The IVC was dissociated from close to the liver to the level of the right renal vein, and also dissociated from below the level of the left renal vein for 2–3 cm. Due to the firm adhesion of the IVC with the abdominal aorta, the IVC was gently dissociated flush against the abdominal aorta to avoid disrupting its thin wall. The left common iliac artery was identified by following the abdominal aorta distally, and was dissociated for 1–2 cm. A 9–0 silk suture was used to temporarily block blood flow from the 2 ends of the dissociated vessels. After the donor liver was placed in the right paracolic sulcus, the recipient left common iliac artery was tied using slip knots with preset 9–0 silk suture, and a wedge incision was made in the segment between the 2 slip knots. End-to-side anastomosis of the donor liver PV and the recipient left common iliac artery (Fig. 1B) was made using a running stitch of 10–0 prolene suture, and the free flap was embedded in the anastomosis to prevent blood flow at an excessive speed or at an excessive volume to lessen high perfusion injury of the donor liver. Then, an end-to-side anastomosis was performed between the donor liver supra-hepatic and infra-hepatic caval vein and the recipient IVC (Fig. 1C, D). The slip knots on the IVC were loosened to allow blood flow to resume. Then, the slip knots on the left common iliac artery were intermittently loosened. The anhepatic phase of the donor liver was considered to be over when pulsations of the reconstructed PV and hepatic artery were visible. A choledochojejunostomy was then performed. Intra-abdominal irrigation with warm normal saline was done for rapid recovery of temperature, and antibiotics were given postoperative as a prophylactic measure.

### Establishing the PVA model in the control group

The surgical steps for extracting the donor liver in the control group were essentially the same as described for the experimental group, with the following exceptions. The donor supra-hepatic caval vein was sutured and closed, and the first PV tributary was ligated. After the recipient right kidney was exposed, the right renal artery and renal vein were dissociated at the renal hilum, and transected. The right ureter and the right adrenal gland vessels were also ligated. After dissociation of the renal fascia, the recipient right kidney was excised. Then, an end-to-end anastomosis of the recipient right renal vein and the donor liver infra-hepatic caval vein was performed using a single running suture. Thereafter, an end-to-end anastomosis was performed of the right renal artery and the donor liver PV using the stent method (Fig. 1E). A choledochojejunostomy was then performed, and after irrigation with warm normal saline the abdomen was closed in layers.

### Postoperative care

All rats received standard postoperative care including ambient warming, electrolyte infusion, and high glucose supplementation. Cox-2 inhibitors were used for postoperative pain control. All rats were weighed on postoperative day (POD) 1, 3, 5, 7, and 14. The rats overall condition and food intake were monitored.

### Biochemical indices

On POD 1, 3, 5, 7, and 14, 5 mL of blood was withdrawn from the rats via the abdominal aorta, and centrifuged at 3500 rpm/min for 10 min. The plasma levels of alanine aminotransferase (ALT), aspartate aminotransferase (AST), total bilirubin, and cholinesterase were determined.

### Histological examination of liver tissues

Rats were sacrificed on POD 1, 3, 5, 7, and 14 by intraperitoneal injection of barbital. Donor liver tissues were obtained and fixed in 10% formaldehyde, paraffin embedded and sectioned (5-μm thick), stained with hematoxylin and eosin (HE), and examined using an inverted light microscope and photographed.

### Immunohistochemistry analysis

Liver tissue sections were dewaxed, hydrated, and washed. After neutralization with 3% endogenous peroxidase and microwave antigen retrieval, slides were preincubated with blocking serum and then incubated overnight with antibodies against tumor necrosis factor (TNF)-α (ab199013, dilution 1:200), interleukin (IL)-6 (ab9324, dilution 1:200), and hepatocyte growth factor (HGF) (ab83760, dilutions 1:200) (Abcam). Three images of 5 representative fields were captured using a Canon EOS600D camera connected to a microscope at a magnification of × 200. Images were analyzed with Image-Pro Plus version 6.2 software (Media Cybernetics), using a special function called “measurement of integrated absorbance”. The integrated absorbance of positive staining of TNF-ɑ, IL-6, and HGF in each photograph was measured, and its ratio to total area of each photograph was calculated as density. The average integrated absorbance value (integrated absorbance/total area) of each slide (3 images) was used to represent a particular sample.

### Immunoblotting assays

Liver tissues were homogenized and lysed in RIPA containing PMSF (Beyotine). The cellular lysates were clarified by centrifugation at 12,000 rpm for 15 min. Protein concentration in the lysate was determined using the Bradford method. Immunoblotting assays were performed using a standard protocol. Protein samples were resolved by sodium dodecyl sulfate polyacrylamide gel electrophoresis (SDS-PAGE). Thereafter, the proteins were transferred onto PVDF membranes. After blocking in 5% non-fat milk, the membranes were probed with primary antibodies against TNF-ɑ (ab199013, dilution 1:1000), IL-6 (ab9324, dilution 1:1000), and HGF (ab83760, dilution 1:1000) (Abcam) and GADPH (EMM0215, dilution 1:1000; Elabscience). Protein bands were visualized using an enhanced chemiluminescence method, and analyzed using ImageJ software. Protein expression was normalized against GADPH. The gray ratio of the target bands to that of the internal reference bands was considered the relative expression of the target proteins.

### Statistical analysis

When data were normally distributed, means were compared between groups. Any 3 of the following 4 variables (effect size, power, confidence level, and sample size) are enough for the calculation of the remaining variable. In the present study, the effect size, power, and confidence level were pre-defined, and then the sample size was calculated (as in similar studies).

We assumed a statistical power of 0.8 at a confidence level of 0.95, and the effect size between the experimental group and the control group was set at a minimum of 0.9.

The formula used to calculate the sample number using the aforementioned variables was:

$$n = \frac{{\left( {\sigma_{1}^{2} + \sigma_{2}^{2} } \right)\left( {Z_{1 - \alpha /2} + Z_{1 - \beta } } \right)^{2} }}{{\left( {\mu_{1} - \mu_{2} } \right)^{2} }}$$

Based on the calculation, a total of 27 rats in each group were required to meet the aforementioned parameters. Assuming a 10% failure rate of the animal experiments, we included 30 rates in each group. Data were expressed as mean ± standard deviation, and Student’s t test was used to compare data of the 2 groups. Statistical analysis was performed using SPSS version 22.0 software (SPSS Inc., Chicago, IL, USA). In all analysis, a value of P < 0.05 was considered to be statistically significant.

## Results

### Changes of liver function

Two rats in the control group and 1 rat in the experimental group died due to intraoperative bleeding, and were not included in the analysis. All the other rats resumed activity 1 to 2 h postoperatively, and started drinking and eating.

**Fig. 2:**
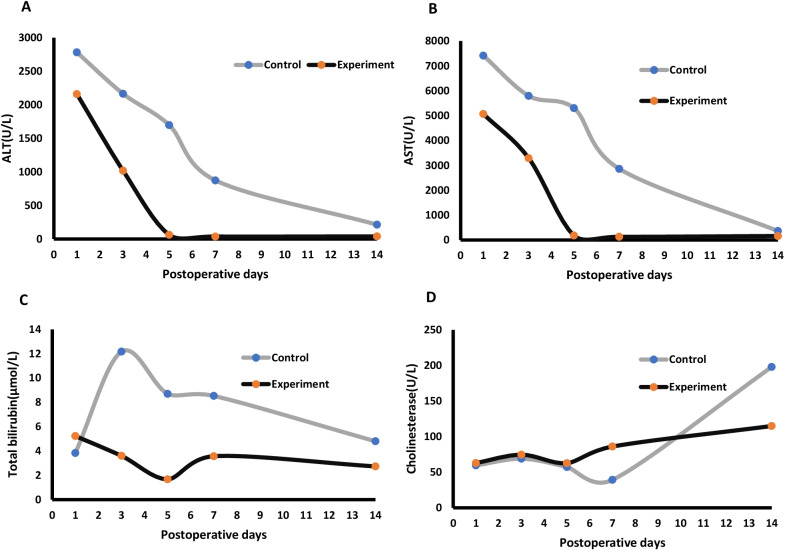
Temporal changes in the plasma concentrations of liver function parameters post-transplant. **A** ALT; **B** AST; **C** total bilirubin; **D** cholinesterase. Data are expressed as mean ± standard deviation. *P < 0.05 vs*.* the control group

On POD 1, there was a marked increase of plasma ALT concentration in both the control group and the experimental group. The ALT plasma concentration in the experimental group rapidly declined beginning POD 3 and reached the normal range on POD 5. The ALT plasma concentration of the control group steadily declined from POD 3, but was still elevated on POD 14 (Fig. 2A). Similarly, the plasma concentration of AST declined more rapidly in the experimental group than the control group after an initial increase on POD 1 (Fig. 2B). On POD 14, the plasma concentration of AST had returned to normal in the experimental group, but remained elevated in the control group (*P* < 0.05). Total bilirubin concentration also steadily declined and was within the normal range on POD 14 in the experimental group, while it remained elevated from POD 1 to 14 in the control group (Fig. 2C). In addition, cholinesterase concentration was significantly higher in the control group than the experimental group on POD 14 (*P* < 0.05) (Fig. 2D).

### Histological changes

#### Experimental group histological findings

Light microscopy of HE stained tissue showed the hepatic lobules, central veins, and portal areas were clear, the central veins exhibited mild dilation and congestion, and there was no obvious infiltration of inflammatory cells. The surrounding sinusoids displayed dilation and congestion, the volume and nuclei of sinusoidal endothelial cells were increased, and their cytoplasm was red-stained. Some hepatocytes were reduced in size, had nuclei that were shrunk with nuclear pyknosis and necrosis, the cytoplasm was red-stained in the portal area, and the cell volume was increased. Binuclear regenerated hepatocytes were clearly observed, a small number of inflammatory cells were observed in the portal area, and interlobular veins showed mild dilatation and congestion.

#### Control group histological findings

**Fig. 3:**
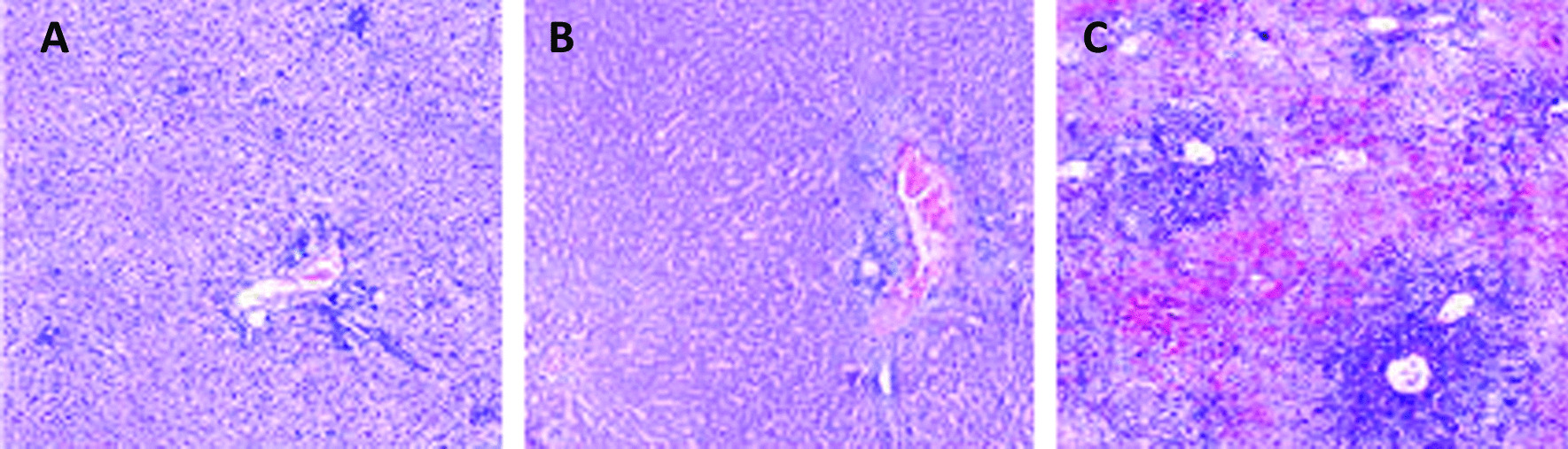
Hematoxylin and eosin staining of normal rat liver (**A**), and donor liver 14 days post-transplant in the experimental group (**B**) and the control group (**C**). Magnification × 40

Light microscopy examination of HE stained tissue specimens showed disordered hepatic lobules, central veins, and portal areas, with obvious infiltration of inflammatory cells. The sinusoidal endothelial cells were enlarged with enlarged nuclei, and the cytoplasm was lightly-stained. The hepatocytes were reduced in size and had shrunken nuclei with clearly observable nuclear pyknosis and necrosis. The cytoplasm was red-stained, and the cell volume increased in the portal area. There was a small number of regenerated hepatocytes, a large number of inflammatory cells in the portal area, and the interlobular veins were dilated and congested (Fig. 3A–C).

### TNF-α, IL-6, and HGF expression in liver tissues

**Fig. 4:**
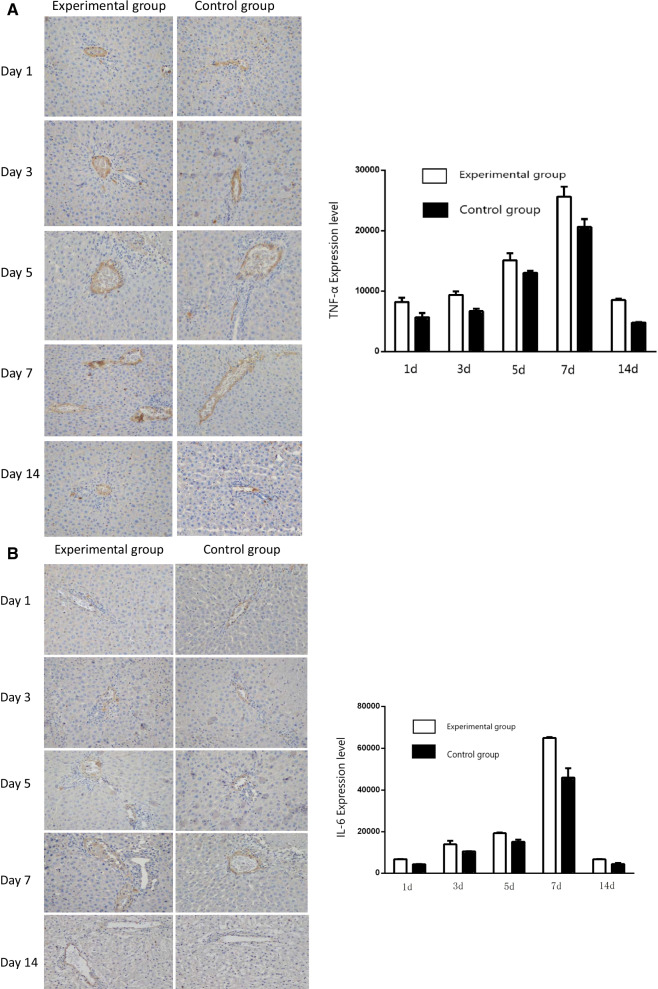

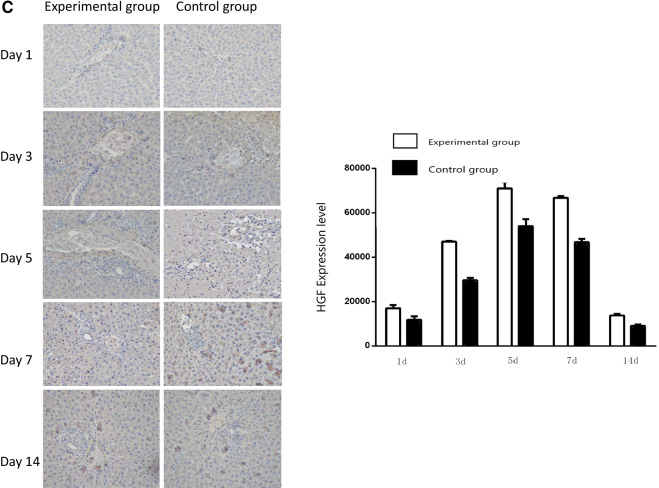
Immunohistochemical staining of liver tissue with specific antibodies. **A** Expression of TNF-α in liver tissue on post-transplant day 1, 3, 5, 7, and 14 in the experimental group and the control group (left panel). **B** Expression of IL-6 in liver tissue on post-transplant day 1, 3, 5, 7, and 14 in the experimental group and the control group (left panel). **C** Expression of HGF in liver tissue at post-transplant day 1, 3, 5, 7 and 14 in the experimental group and the control group (left panel). In **A**, **B**, and **C** expression is quantified and shown in bar graphs (right panels). Magnification × 200

**Fig. 5:**
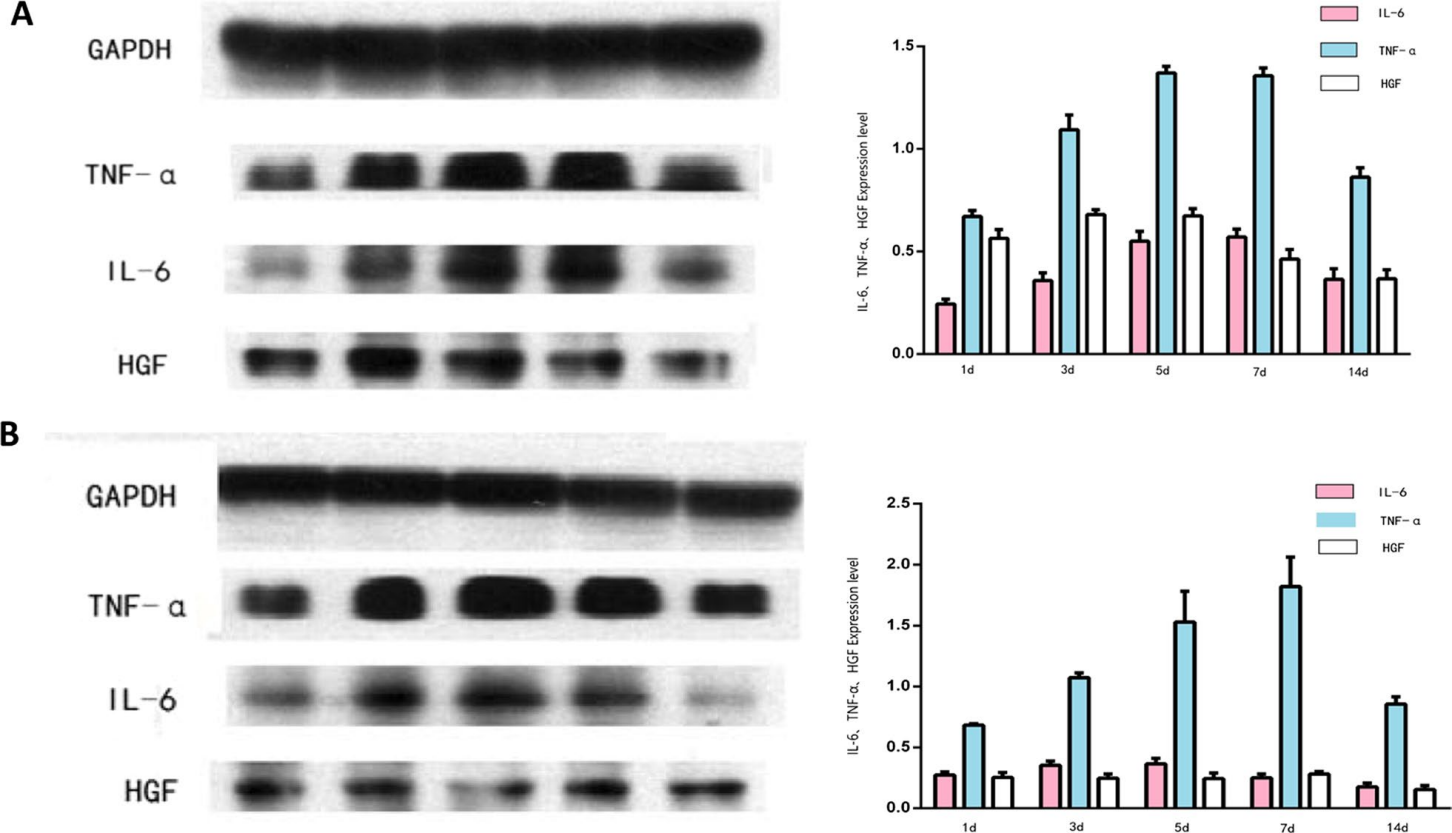
Immunoblotting assays showing the expression of TNF-α, IL-6, and HGF in liver tissue on post-transplant day 1, 3, 5, 7, and 14 (left panels). **A** Experimental group. **B** Control group

The expression of TNF-α in liver tissues was examined by immunohistochemistry and immunoblotting assays. Immunohistochemical staining revealed that TNF-α levels steadily increased in liver tissue in both the control group and the experimental group from POD 1, peaked on POD 7, and subsequently declined to POD 14. TNF-α levels in the liver tissue of the experimental group were significantly higher than those in the control group at all time points examined (*P* < 0.05) (Fig. 4A). IL-6 levels in liver tissue exhibited similar changes to that of TNF-α, with a steady increase in both groups from POD 1 to a peak on POD 7 (Fig. 4B). HGF levels in liver tissue steadily increased in both the control group and the experimental group from POD 1 to a peak on POD 5, and thereafter declined in both groups. HGF levels in the liver tissue of the experimental group were significantly higher than those in the control group at all time points examined (*P* < 0.05) (Fig. 4C). The aforementioned findings were confirmed by immunoblotting assays (Fig. 5).

## Discussion

The results of this study showed that the modified, kidney-sparing PVA model of heterotopic auxiliary liver transplantation is more conducive to liver regeneration with quicker return of liver function. The method is associated with less inflammatory cell infiltration and less hepatocyte necrosis than the more common method it was compared to. Greater success with auxiliary liver transplantation will help to improve the shortage of donor livers.

Cohn et al. [12] conceived the idea of liver perfusion using arterial blood instead of blood from the PV in 1952. Their studies showed that normal liver blood flow could be maintained by perfusing the liver with arterial blood with the right flow rate and pressure through the PV, thus maintaining liver function and promoting liver regeneration. This was considered a novel approach for addressing the shortage of donor livers for transplantation. Subsequently, in 1955 Welch et al. [13] conceived the idea of auxiliary liver transplantation, which provided a theoretical basis for subsequent studies on liver transplantation and donor liver regeneration. In 1998, Erhard et al. [14] developed PVA in heterotopic auxiliary liver transplantation to reduce the risk of donor liver PV thrombosis, and Margarit et al. [15] used the method to treat fulminant hepatic failure in 2000. More recent studies have shown that PVA promotes initial liver regeneration, as well as long-term liver growth [16]. Shimizu et al. [17] found that PVA markedly increased the oxygen saturation of the perfused liver and greatly reduced energy demands, ultimately promoting hepatocyte regeneration. Other studies have also confirmed the positive effect of PVA on liver regeneration [18, 19]

Most current studies regarding PVA in heterotopic auxiliary liver transplantation have placed the donor liver in the location of a resected kidney [3, 6, 7]. However, this method using end-to-end anastomosis of the renal artery and the PV unnecessarily sacrifices one kidney, and furthermore does not follow normal anatomic and physiological relations. In the current study, we established a novel PVA model by placing the donor liver in the right paracolic sulcus, and performed end-to-side anastomosis of the donor liver PV and recipient left common iliac artery. Our results showed that as compared to the more conventional method, our modified PVA method was associated with an appropriate blood perfusion volume, velocity, and pressure; thus, lessening the risks of excessive congestion and rupture of the hepatic capsule [20]. Furthermore, the anhepatic phase was shortened due to the abundance of oxygenated arterial blood supplied in a relatively short time. Studies have shown that an increased blood flow volume and enhanced oxygen supply after PVA is conducive to liver regeneration [21–23]. Other studies have further revealed that PVA has no effect on normal liver function, but promotes energy metabolism and recovery of hepatic reserve function [24–26]. In the current study, we only used 30% donor liver; a much lower volume than typically used. Fan et al. [27] reported that PVA increases oxygen transport to the donor liver by increasing the extent of liver resection and improving the rate of regeneration of the remnant liver. Other studies have also confirmed the positive effects of PVA on liver regeneration [17, 21, 28–31].

Liver regeneration is a complex process that requires multiple cellular factors, and is mainly initiated by the release of cytokines, and IL-6, TNF-α, and HGF are important modulators of liver regeneration [32, 33]. Notably, when the liver returns to approximately its normal size regeneration ceases [5]. IL-6 is produced by macrophages in the liver and hepatocytes, and activates Kupffer cells to release TNF-α, which in turn stimulates IL-6 production [5, 32, 33]. TNF-α and IL-6 are the main modulators of liver regeneration, and induce hepatocyte proliferation by activating NF-κβ and STAT3 [32, 33]. In addition, lipopolysaccharides C3a and C5a bind to their respective receptors on macrophages in the liver, and play a role in liver regeneration by modulating TNF-α and IL-6 expression [33, 33]. Study has shown that IL-6/sIL-6R in combination with growth factors promote initiation of the hepatocyte cell cycle via PI3K/AKT signaling to promote liver regeneration [34]. It has also been reported that TNF-α and epidermal growth factor (EGF) promote DNA replication during liver regeneration [35]. Study has also shown that natural killer (NK) cells and NK-T cells upregulate TNF-α and IL-6/STAT3 via an immune response, and together with HGF promote liver regeneration [36].

HGF is an important growth factor for hepatocytes, and accelerates cell cycle progression during liver regeneration. HGF is produced by mesenchymal cells and acts on hepatocytes in a paracrine and endocrine manner. HGF is considered an initiator of liver regeneration, and is directly mitogenic for hepatocytes as well as protective of hepatocytes [32, 33]. HGF is upregulated during liver regeneration and peaks at day 7 post liver transplant [5].

Our immunohistochemistry analysis showed that the expression of TNF-α and IL-6 was markedly increased on POD 1 in both groups, and peaked on POD 5 and 7, respectively, and thereafter declined. These findings indicate that liver regeneration is initiated after partial hepatectomy and PVA. Notably, the levels of TNF-α, and IL-6, and HGF in the liver tissue of the experimental group were markedly higher than in the control group postoperatively. Furthermore, histological examination of liver tissue showed less inflammatory cell infiltration and greater hepatocyte regeneration in the experimental group than the control group. Also noteworthy, postoperatively indices of liver function (e.g., ALT) decreased more rapidly in the experimental group than the control group. Taken together, these results suggest the modified method of axillary liver transplantation we studies promotes liver regeneration to a greater degree than the more common method it was compared to.

Also noteworthy, the mortality rate of rats in the control group was higher than that of the experimental group. The exact reason is unknown, but could be due to postoperative hemorrhage or PV thrombosis. However, most importantly, the modified procedure we have described is associated with lower mortality than the more conventional procedure it was compared to.

The primary limitation of this study is that it was performed using an animal model. However, the results suggest that the method provides for greater liver regeneration and that human trials may be warranted.

## Conclusions

The modified PVA model of heterotopic auxiliary liver transplantation examined in this study promotes liver regeneration than the more conventional method it was compared to. The modified model was associated with more favorable histological changes in liver tissue, faster recovery of markers of liver function (e.g., AST), higher postoperative levels of markers of liver regeneration (TNF-α, IL-6, HGF), and a lower mortality rate. Notably, only 30% of a donor liver was sufficient for recovery of the recipient liver, which may help the marked shortage of donor livers. While this was an animal study, the positive results suggest that human trials may be warranted.

## Data Availability

The datasets used and/or analyzed during the current study are available from the corresponding author on reasonable request.
